# Research and Optimization of White Blood Cell Classification Methods Based on Deep Learning and Fourier Ptychographic Microscopy

**DOI:** 10.3390/s25092699

**Published:** 2025-04-24

**Authors:** Mingjing Li, Junshuai Wang, Shu Fang, Le Yang, Xinyang Liu, Haijiao Yun, Xiaoli Wang, Qingyu Du, Ziqing Han

**Affiliations:** College of Electronic Information Engineering, Changchun University, Changchun 130022, China; limj@ccu.edu.cn (M.L.); 240401173@mails.ccu.edu.cn (J.W.); 230402191@mails.ccu.edu.cn (L.Y.); 230402186@mails.ccu.edu.cn (X.L.); yunhj@ccu.edu.cn (H.Y.); wangxli@ccu.edu.cn (X.W.); 230401175@mails.ccu.edu.cn (Q.D.); 230401178@mails.ccu.edu.cn (Z.H.)

**Keywords:** white blood cell classification, Fourier ptychographic microscopy, YOLOv7, CARAFE upsampling, EMA mechanism, Soft-NMS

## Abstract

White blood cell (WBC) classification plays a crucial role in hematopathology and clinical diagnostics. However, traditional methods are constrained by limited receptive fields and insufficient utilization of contextual information, which hinders classification performance. To address these limitations, this paper proposes an enhanced WBC classification algorithm, CCE-YOLOv7, which is built upon the YOLOv7 framework. The proposed method introduces four key innovations to enhance detection accuracy and model efficiency: (1) A novel Conv2Former (Convolutional Transformer) backbone was designed to combine the local pattern extraction capability of convolutional neural networks (CNNs) with the global contextual reasoning of transformers, thereby improving the expressiveness of feature representation. (2) The CARAFE (Content-Aware ReAssembly of Features) upsampling operator was adopted to replace conventional interpolation methods, thereby enhancing the spatial resolution and semantic richness of feature maps. (3) An Efficient Multi-scale Attention (EMA) module was introduced to refine multi-scale feature fusion, enabling the model to better focus on spatially relevant features critical for WBC classification. (4) Soft-NMS (Soft Non-Maximum Suppression) was used instead of traditional NMS to better preserve true positives in densely packed or overlapping cell scenarios, thereby reducing false positives and false negatives. Experimental validation was conducted on a WBC image dataset acquired using the Fourier ptychographic microscopy (FPM) system. The proposed CCE-YOLOv7 achieved a detection accuracy of 89.3%, showing a 7.8% improvement over the baseline YOLOv7. Furthermore, CCE-YOLOv7 reduced the number of parameters by 2 million and lowered computational complexity by 5.7 GFLOPs, offering an efficient and lightweight model suitable for real-time clinical applications. To further evaluate model effectiveness, comparative experiments were conducted with YOLOv8 and YOLOv11. CCE-YOLOv7 achieved a 4.1% higher detection accuracy than YOLOv8 while reducing computational cost by 2.4 GFLOPs. Compared with the more advanced YOLOv11, CCE-YOLOv7 maintained competitive accuracy (only 0.6% lower) while using significantly fewer parameters and 4.3 GFLOPs less in computation, highlighting its superior trade-off between accuracy and efficiency. These results demonstrate that CCE-YOLOv7 provides a robust, accurate, and computationally efficient solution for automated WBC classification, with significant clinical applicability.

## 1. Introduction

With the rapid advancement of optical imaging technology and deep learning, white blood cell classification has gained increasing importance in hematopathology and clinical diagnosis. Numerous clinical studies have demonstrated that pathological changes are often accompanied by alterations in the quantity and morphology of blood cells, providing critical evidence for early disease diagnosis [1]. Traditional classification methods primarily rely on manual microscopic examination or automated blood analyzers. While manual analysis is susceptible to subjectivity and low efficiency, blood cell analyzers—though capable of improving detection speed—still fall short in terms of morphological classification accuracy. Additionally, conventional image processing-based methods depend on fixed rules, making them inadequate for coping with the diverse and complex morphological variations of white blood cells. Deep learning techniques, though superior in feature learning, often face challenges such as limited receptive fields, high computational complexity, and contextual information loss, which restrict their effectiveness in clinical applications.

To address these limitations, YOLOv7 has emerged as a powerful deep learning-based object detection algorithm with superior accuracy and real-time performance. It has been widely adopted in medical image analysis due to its ability to extract more detailed image features compared to other algorithms. Structurally, YOLOv7 consists of three main components: the input layer, the backbone network, and the detection head [2]. The input module resizes images to 640 × 640 × 3, which are then processed through a series of CBS modules, ELAN modules, and MP modules in the backbone to extract features. In the prediction stage, feature MAPs from multiple scales are generated via FPN (Feature Pyramid Network) and PAN (Path Aggregation Network), followed by multi-scale prediction and final detection results through Non-Maximum Suppression (NMS). Despite these advantages, YOLOv7 still faces difficulties in balancing model accuracy, parameter count, and computational efficiency. Specifically, the traditional YOLOv7 network may encounter challenges such as insufficient global context modeling, suboptimal upsampling quality, and limitations in detecting densely packed or overlapping white blood cells.

In light of these challenges, this paper proposes an improved white blood cell classification algorithm—CCE-YOLOv7—based on the YOLOv7 framework. The proposed algorithm incorporates a series of targeted enhancements: (1) the Conv2Former backbone, which integrates convolutional operations with Transformer-based attention to improve global context modeling; (2) the CARAFE upsampling operator, which employs content-aware mechanisms to reconstruct high-resolution features and expand the effective receptive field; (3) the Efficient Multi-scale Attention (EMA) module, which enhances multi-scale feature fusion and focuses more effectively on key regions; and (4) an optimized prediction strategy using Soft-NMS, which reduces false detections in dense object areas. These improvements are herein validated through ablation and comparative experiments, demonstrating significant gains in accuracy and computational efficiency over the original YOLOv7 and providing a robust and efficient solution for automated white blood cell classification in clinical practice.

## 2. Related Works

### 2.1. Imaging Technology

#### 2.1.1. Introduction to Fourier Microimaging

Fourier ptychographic microscopy (FPM) technology provides a groundbreaking solution for traditional white blood cell detection, which primarily relies on manual observation under a microscope and automated blood cell analyzers. The former requires high operational expertise and is prone to subjective influences, while the latter still requires manual confirmation when the equipment malfunctions, making it difficult to simultaneously meet the demands for high resolution and large field-of-view imaging [3].

FPM technology captures low-resolution images using a low-magnification objective lens and significantly extends the system’s space-bandwidth product (SBP) through the fusion and reconstruction of frequency-domain information. This allows FPM to maintain the advantages of a large field of view and long focal depth while reconstructing high-resolution and quantitative phase images. This capability enables FPM not only to comprehensively display the overall distribution of white blood cells but also to capture subtle morphological features, providing more precise imaging support for white blood cell classification and pathological diagnosis.

Researchers worldwide have made significant progress in advancing the application of FPM technology for white blood cell detection. In 2013, Zheng et al. [4] first introduced Fourier ptychographic microscopy, laying the theoretical foundation for achieving both high-resolution and large-field-of-view imaging. In 2017, Rodríguez et al. [5] implemented Fourier ptychographic single-pixel imaging, which enhanced the system’s flexibility. In 2018, Konda et al. [6] proposed multi-aperture Fourier synthetic microscopy, further enhancing imaging capabilities through oblique illumination and target arrays. In 2019, Song et al. [7] developed a fast Fourier transform phase recovery scheme, speeding up the phase reconstruction process. In 2021, Wei et al. [8] proposed a method for LED (Light Emitting Diode) position error correction, improving reconstruction accuracy. In 2022, Zhu et al. [9] introduced a spatial domain correction method based on a particle swarm optimization algorithm, effectively addressing LED array installation errors. Additionally, scholars such as Lee [10], Zhou [11], Sun [12], and Shu [13] have explored areas such as single-shot imaging, wavelength multiplexing, color multiplexing, and circular illumination, significantly improving data acquisition efficiency and imaging quality and offering robust support for the application of FPM technology in white blood cell detection and pathological analysis.

In summary, Fourier ptychographic microscopy, with its distinct advantages of both high resolution and a large field-of-view, has opened up new application prospects for white blood cell detection and related pathological analysis. With the ongoing refinement of system parameters, enhanced data acquisition efficiency, and integration with cutting-edge technologies such as deep learning, FPM is expected to further surpass existing imaging limitations, offering more precise and efficient technical support for clinical diagnosis and scientific research.

#### 2.1.2. Leukocyte Dataset

To achieve high-quality white blood cell (WBC) image acquisition, precise calibration of the LED array illumination system must be performed prior to imaging. Initially, the central LED of the array was carefully aligned with the optical aperture of the microscope to minimize stray light interference and ensure uniform and focused illumination. Subsequently, a 4× objective lens was accurately aligned with the microscope tube to guarantee stable light transmission and precise image formation. Before imaging, the lens surface was thoroughly cleaned using alcohol swabs to remove dust and potential contaminants, thereby reducing image noise and enhancing clarity. The blood smear sample was then placed on the microscope stage with the target region positioned at the center of the aperture to ensure even and adequate illumination across the field of view. Finally, the LED array was activated and its brightness adjusted to achieve optimal illumination, ensuring that only the LEDs aligned with the optical path provided maximum illumination of the sample, resulting in clear and stable WBC images.

Following sample preparation and imaging setup, raw blood cell images were captured using a Fourier ptychographic microscopy (FPM) system. Due to the high abundance of red blood cells, platelets, and other impurities in peripheral blood, the acquired images often contain substantial background noise and irrelevant information. Additionally, WBCs are relatively scarce and exhibit uneven class distribution across different subtypes. Therefore, during dataset construction, the raw images were cropped to extract regions of interest that conform to the input size requirements of deep learning models. Using automated algorithms, the target regions were localized and cropped into images of 640 × 640 pixels, ensuring no WBC was excluded. This process ultimately yielded 1000 valid blood cell images containing WBCs.

Considering the data-hungry nature of deep learning models and the necessity of diverse sample representations, relying solely on the original image set may lead to overfitting due to insufficient coverage of the full feature space of each WBC subtype. To address this, data augmentation techniques were applied during preprocessing, including image rotation, scaling, translation, and brightness adjustment. These methods effectively increased the dataset size from 1000 to 5000 images and enhanced sample diversity. Particularly for underrepresented WBC classes, such augmentation significantly improved data balance. The augmented images exhibited greater variation in cell size, shape, and orientation, enabling the model to better learn the discriminative features of each WBC type.

To ensure scientific rigor in model training and objective evaluation, the constructed dataset was divided into three subsets: training, validation, and testing. The training set was used for model optimization and parameter learning, the validation set for monitoring model performance and preventing overfitting during training, and the test set—completely independent from the training process—served for final performance evaluation, thereby ensuring the model’s generalization ability.

### 2.2. White Blood Cell Detection

Traditionally, pathologists perform blood smear analysis by manually identifying and counting white blood cells (WBCs). However, this method is labor-intensive, time-consuming, and prone to errors, often influenced by subjectivity [14]. Although existing white blood cell analyzers can provide basic counting information, they still have limitations in capturing the morphological features of white blood cells, which somewhat limits their effectiveness in aiding medical diagnosis. Currently, white blood cell detection primarily relies on two technical approaches: one based on traditional image processing techniques and the other utilizing deep learning technology for automated analysis. Due to the rapid advancements in computer vision and digital technologies, the integration of deep learning and computer graphics methods has provided clinicians with new auxiliary tools, making the detection of white blood cell quantity and morphology more precise. This progress has not only improved detection efficiency and result consistency but also offers more reliable data support for early disease diagnosis and treatment.

#### 2.2.1. White Blood Cell Classification

**Figure 1:**
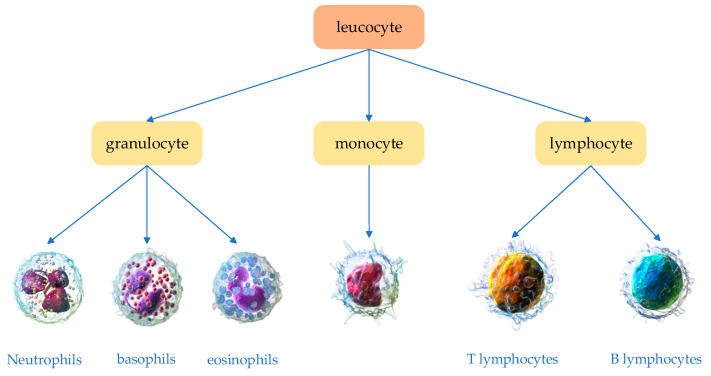
Classification of White Blood Cell Subtypes.

White blood cells are the core defense of the human immune system, primarily protecting the body by eliminating pathogenic microorganisms such as bacteria and viruses. During this process, different subtypes of white blood cells—including monocytes, lymphocytes, and granulocytes—each play a unique role. Notably, after staining, granulocytes can be further classified into neutrophils, basophils, and eosinophils (see Figure 1). These classifications provide essential reference information for evaluating immune status and pathological changes. In contrast, although red blood cells account for 93% to 96% of the blood, their detection is mainly used to aid in the diagnosis of blood disorders such as iron deficiency anemia and vitamin B12 deficiency. Changes in white blood cells, however, hold greater clinical significance in reflecting the body’s immune response.

Under normal conditions, the concentration of white blood cells typically remains within a relatively stable range, reflecting the body’s normal immune function. In the presence of infection or other pathological conditions, the total white blood cell count and the distribution of its subtypes can be significantly altered, providing critical information for clinical diagnosis. As shown in Table 1, fluctuations in the quantities of different types of white blood cells correspond to specific pathological implications. For example, acute infections are often associated with a significant increase in neutrophils, while certain immunosuppressive conditions may lead to a decrease in lymphocyte count.

Thus, exploring the relationship between white blood cell count, subtype distribution, and diseases not only helps improve diagnostic accuracy but also provides scientific evidence for personalized treatment plans. Monitoring the dynamic changes in white blood cells plays a crucial role in early clinical warning and disease management. A white blood cell classification algorithm based on CCE-YOLOv7 is proposed in this paper to address the specific requirements of white blood cell classification, eliminating the need for complex image segmentation and feature extraction processes while directly leveraging deep learning’s ability to classify white blood cells. In deep learning-based white blood cell classification methods, accurate classification is achieved by analyzing the morphological, color, and texture differences and characteristics between white blood cells. The core of this method lies in using deep learning models to learn and recognize these features from a large amount of training data. Since basophils constitute a very small proportion of the total white blood cell count in blood, obtaining a sufficient number of basophil images for data training and model optimization presents a significant challenge in practical applications. Given this challenge, the classification of neutrophils, lymphocytes, monocytes, and eosinophils is primarily focused on in this paper, with the characteristics of each white blood cell type presented in Table 2.

#### 2.2.2. Image Processing-Based Methods

In recent years, significant progress has been made in white blood cell detection based on image processing methods. However, these methods still have some limitations in practical applications. For example, the differential evolution (DE) algorithm proposed by Cuevas requires optimization encoding of candidate ellipses to adapt to the white blood cell morphology in edge-mapped images [16]. This method is computationally intensive and highly dependent on the quality of image preprocessing. Kasim employed a mixed-space learning structure combining K-means clustering and expectation maximization to locate the region of interest, aiming to reduce the impact of staining and lighting variations on detection [17]. However, this method may not consistently and accurately locate white blood cell regions when dealing with images with uneven staining or severe lighting changes. Cheng proposed a fuzzy morphological neuron model network, which converts the image from the RGB color space to the HSL color space and then uses a fuzzy morphological network for white blood cell recognition [18]. This method may introduce information loss during the color space conversion, and the parameter settings of the fuzzy morphological network have a significant impact on the recognition results. Lin et al. proposed a white blood cell extraction algorithm based on feature-weighted adaptive K-means clustering, combining color space decomposition, K-means clustering, and watershed algorithms for image segmentation and classification [19]. This method has a complex process, large computational load, and is sensitive to parameter selection. Overall, these methods may face issues in practical applications, such as high computational complexity, strong dependency on preprocessing, and sensitivity to parameter settings, which limit their promotion and application in clinical diagnosis.

#### 2.2.3. Deep Learning-Based Approach

In recent years, deep learning technology has witnessed extensive application in the field of white blood cell detection, with related research concentrating on three principal directions. The first category comprises CNN (convolutional neural network)-based classification methods, which utilize CNN models to extract features from white blood cell images and classify them through deep neural networks. For example, Wang Xuetong et al. proposed a CNN model incorporating attention mechanisms, attaining significant progress in enhancing white blood cell classification accuracy [20]. Similarly, Siddique et al. [21] introduced an improved deep learning-based classification model for white blood cell images, demonstrating superior performance in classifying different cell types using enhanced network structures. Nevertheless, such methods generally rely on substantial amounts of labeled data and have limited capability in modeling within-feature space relationships, which may result in misclassification, particularly in tasks concerning white blood cells with complex morphologies.

The second category consists of object detection-based recognition methods, which employ object detection algorithms to locate and classify white blood cells. For instance, Sun C. et al. achieved precise detection and counting of white blood cells predicated on the Faster R-CNN (Region-based Convolutional Neural Network) model [22]. In contrast to CNN classification methods, these approaches can directly predict the target location and category, thereby enhancing detection efficiency. However, due to the high computational complexity of models like Faster R-CNN, problems with slower inference speed may emerge in practical applications, especially in scenarios demanding real-time detection.

The third category encompasses integrated methods that combine diverse features, integrating deep learning with morphological features to boost the robustness of white blood cell detection [23]. For example, the model proposed by Yuzhuo Chen et al., DAFFNet (Deep Aggregation of Feature Fields Network), integrates high-level semantic features with morphological features, achieving more precise white blood cell classification. Additionally, Khan and Mir R. [24] presented a hybrid approach that employs U-Net CNN for accurate white blood cell segmentation and utilizes hand-crafted features for effective classification. This approach enhances model interpretability and generalization ability by incorporating prior domain knowledge from traditional feature engineering. However, it involves complex feature fusion strategies, which can lead to higher computational overhead and still require further optimization for adaptation across different datasets.

A detection method based on the YOLO (You Only Look Once) architecture is employed in this paper.

## 3. Method

### 3.1. CCE-YOLOv7 Network Architecture

**Figure 2:**
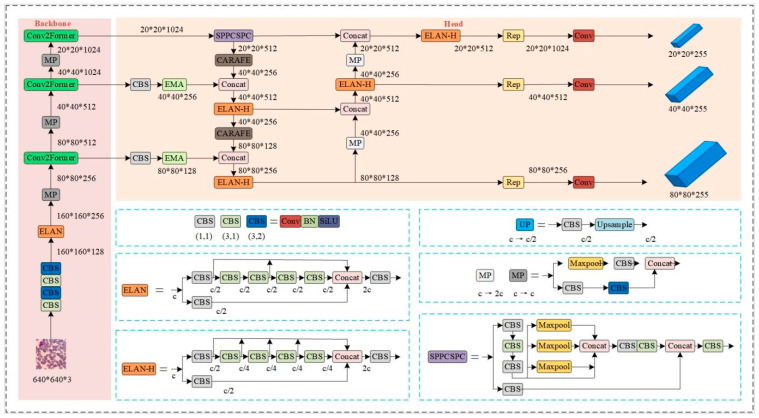
The network structure of CCE-YOLOv7.

Traditional methods typically extract feature information from white blood cell images through segmentation. However, due to significant color differences within the same type of white blood cells as well as morphological variations and blurred features between different cells, classification becomes more challenging. Moreover, classification performance is directly influenced by the quality of segmentation. To address these challenges, a white blood cell classification algorithm based on CCE-YOLOv7 is proposed in this paper, significantly enhancing classification performance. The network architecture is illustrated in Figure 2.

First, the input image is resized to 640 × 640 × 3 before passing through several CBS (Convolutional Block Attention) modules, ELAN (Efficient Layer Aggregation Network) modules, and MP (Max Pooling) modules. Additionally, a novel Conv2Former convolutional neural network structure is introduced. This configuration efficiently extracts and processes features, allowing the network to better capture global information and contextual semantic details, thus improving overall performance. Second, a lightweight upsampling operator (CARAFE) is employed to reduce the number of parameters and computational load. The upsampling process benefits from a larger receptive field, which enhances the capture of target feature information and improves the quality of the unsampled feature MAP. Next, between the backbone and feature extraction networks, an efficient multi-scale attention module (EMA) is incorporated. This module improves the model’s ability to capture multi-scale information within the image, thereby strengthening the feature representation of white blood cell positions in the feature MAP, which in turn boosts the model’s classification accuracy. Finally, flexible non-maximum suppression (Soft-NMS) is applied to eliminate redundant candidate boxes, ensuring that only the best detection box is retained. This results in more accurate position predictions and enhances the overall precision of white blood cell classification.

**Figure 3:**
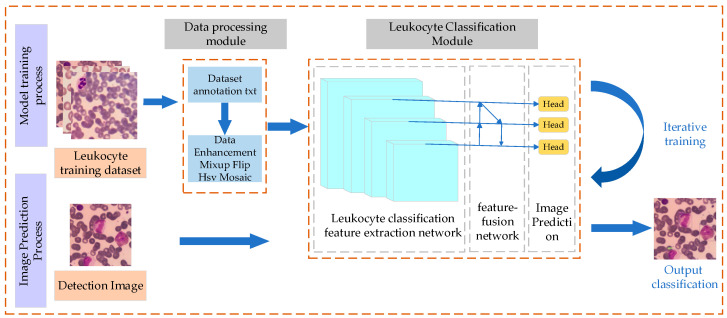
Leukocyte Image Classification Process.

The white blood cell classification module stands as the system’s core component. It primarily takes pre-processed and annotated white blood cell images as input and utilizes the CCE-YOLOv7 model. Through neural network training on a large dataset, the model predicts and classifies white blood cells without requiring additional segmentation processing steps. The white blood cell classification process is illustrated in Figure 3. The entire system is mainly composed of the data preprocessing module and the white blood cell classification module. When compared with the YOLOv7 model, CCE-YOLOv7 has been optimized and improved in several key aspects, including the feature extraction network, feature fusion network, upsampling operator, and non-maximum suppression algorithm. These enhancements significantly boost the improved model’s performance in white blood cell classification.

#### 3.1.1. Conv2Former-Based Backbone Networks

**Figure 4:**
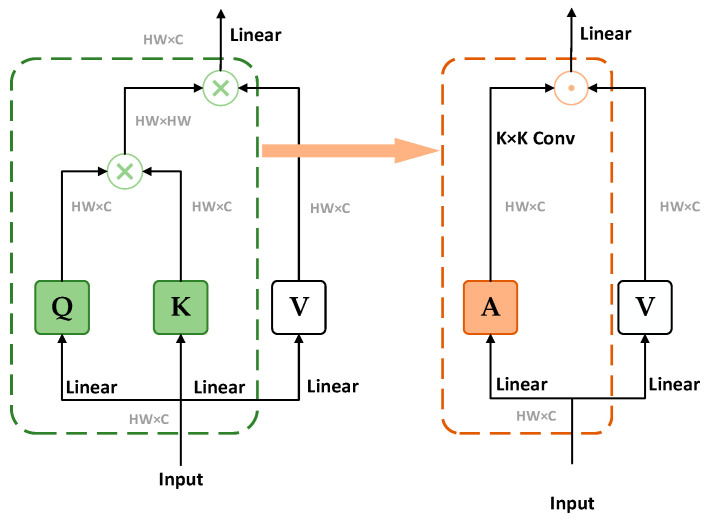
Self-Attention Module with Convolutional Modulation Module.

Since 2020, Vision Transformers (ViTs) [25] have demonstrated exceptional performance in image classification. However, their self-attention mechanism incurs high computational costs when processing high-resolution images. To address this issue, researchers have explored methods that combine convolution with Transformers. The Conv2Former [26], a convolutional transformer, utilizes convolutional modulation operations to significantly reduce computational costs while preserving the advantages of global information modeling, thereby improving the efficiency of high-resolution image processing, as shown in Figure 4.

**Figure 5:**
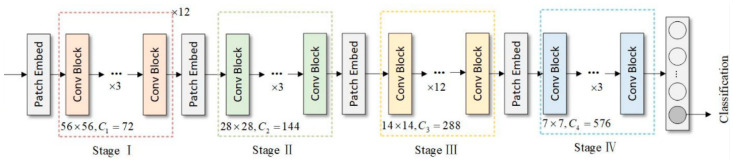
The overall architecture of Conv2Former.

As shown in Figure 5, Conv2Former is a Transformer-convolutional network with a pyramid structure. It consists of four stages, each with a different number of convolution blocks and feature MAP resolutions, with resolution reduced between stages using the Patch Embedding module. This network combines convolution operations with Transformer modules, where the former extracts local features, while the latter captures global semantics. The self-attention mechanism regulates the weight distribution of convolutional feature MAPs, enhancing the representation of multi-scale and multi-directional features. Building on this advantage, certain ELAN modules in the YOLOv7 backbone network were improved, optimizing the balance between network parameters and computational load while enhancing the capture of global information and contextual semantics, thereby significantly improving network performance.

#### 3.1.2. CARAFE Lightweight Upsampling Operator

The nearest neighbor interpolation, as a simple and efficient upsampling method, generates new pixels by directly replicating the neighboring pixels from a low-resolution image. While this approach is computationally fast, it overlooks surrounding pixel information, leading to discontinuous grayscale and jagged edges, which negatively impact YOLOv7’s performance in white blood cell classification tasks. To enhance classification accuracy, the CARAFE [27] algorithm was adopted in this paper to improve the upsampling method. CARAFE is a learnable, content-based upsampling algorithm. It not only leverages context information through a larger receptive field to achieve more precise upsampling but also employs a content-aware sampling technique, combining semantic information from feature MAPs for finer reconstruction. Furthermore, its lightweight structure significantly enhances the resolution and quality of feature MAPs without imposing a substantial computational burden.

**Figure 6:**
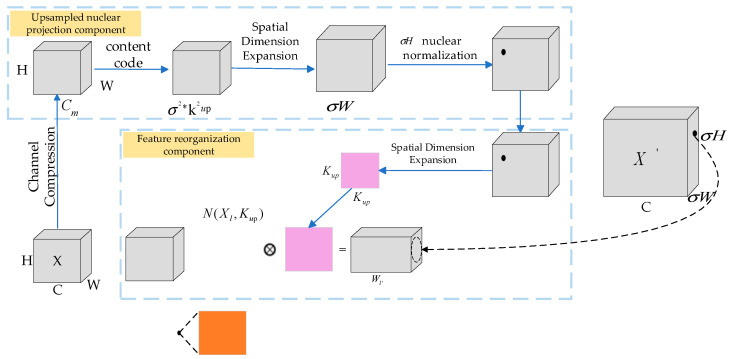
CARAFE upsampling structure.

The CARAFE algorithm consists of two main modules: the upsampling kernel prediction module and the feature reconstruction module (as shown in Figure 6). During the kernel prediction phase, the input feature MAP T undergoes a 1 × 1 convolution to compress the channels, thereby reducing the computational load. The channel is then expanded according to the desired upsampling size and scale, facilitating content encoding. After unfolding the feature MAP in the spatial dimension, Softmax normalization is applied to generate the weights, ensuring the upsampling kernel sums to 1. In the reconstruction phase, the feature MAP is reconstructed by performing a dot product between each corresponding upsampling kernel and the respective location in the feature MAP. This content-aware upsampling technique not only significantly enhances the resolution and quality of the feature MAPs but also mitigates semantic information loss, a common issue with traditional nearest neighbor interpolation, by expanding the receptive field. Consequently, it captures the morphological and shape differences of cell nuclei more accurately in white blood cell classification tasks, thereby optimizing the overall classification performance.

#### 3.1.3. Efficient Multi-Scale Attention Module

The Efficient Multi-scale Attention (EMA) module enhances key features to enable flexible network integration through cross-spatial learning and multi-scale design. This module ensures the even distribution of spatial semantic features by reshaping and grouping channels while encoding global information to calibrate channel weights. Additionally, it aggregates features from different branches through cross-dimensional interactions, thereby improving multi-dimensional perception and multi-scale feature extraction capabilities. During the feature fusion process, the EMA module effectively integrates white blood cell features across different scales, enhancing the feature expression of target regions and boosting the model’s ability to capture critical information. Its parallel multi-scale convolutions accurately represent the white blood cell region features, and the efficient attention mechanism emphasizes classification-related information while suppressing irrelevant redundant features, thereby improving classification performance.

**Figure 7:**
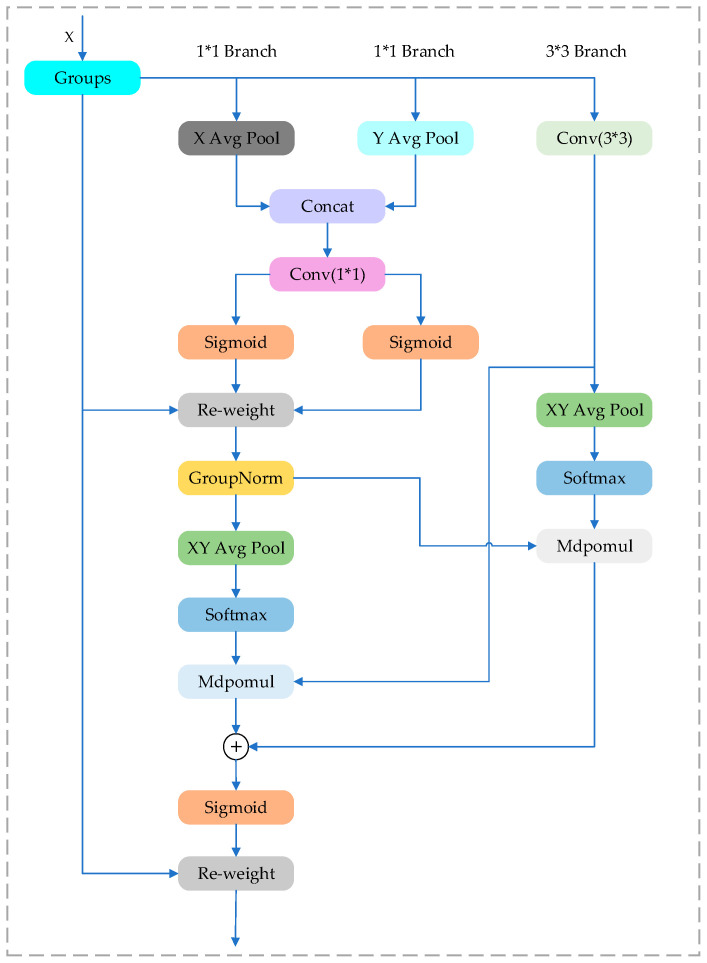
EMA structure.

The architecture of the EMA module is depicted in Figure 7. The input feature map $X\in R^{C\times H\times W}$ is first divided into G groups along the channel dimension:

(1) $$X=\left[X_{1},X_{2},\ldots ,X_{G}\right],X_{g}\in R^{\frac{C}{G}\times H\times W}$$

Each group is processed through parallel attention branches: two 1 × 1 branches and one 3 × 3 branch. The 1 × 1 branches extract global contextual information using global average pooling and 1 × 1 convolution:

(2) $$z_{g}=GAP\left(X_{g}\right)\in R^{\frac{C}{G}\times 1\times 1}$$

Then, it passes through a shared 1 × 1 convolution $W_{C}$ and a Sigmoid activation to generate the channel attention map $M_{c}=\sigma \left(W_{c}*z_{g}\right)$. The 3 × 3 convolutional branch extracts local contextual dependencies $L_{g}={Conv}_{3\times 3}\left(X_{g}\right)$.

Next, two spatial attention maps are computed. The first is generated by applying global average pooling and Softmax normalization $M_{s1}=Softmax\left({GAP}_{2D}\left(M_{c}\right)\right)⊙L_{g}$. The second spatial attention map is derived from element-wise interaction between normalized Mc and encoded Lg $M_{s2}=Norm\left(M_{c}\right)⊙Encode\left(L_{g}\right)$. the two spatial maps are fused and passed through a Sigmoid function and reweighted as $M_{EMA}=\sigma \left(M_{s1}+M_{s2}\right)$ The final output of the EMA module is obtained by reweighting the original input:

(3) $$X^{\prime }=M_{EMA}⊙X$$

This module effectively enhances the expressive power of deep features by leveraging both local and global contextual information through multi-scale convolutions and adaptive attention weighting.

#### 3.1.4. Non-Maximum Value Suppression Optimization

In the task of white blood cell classification, YOLOv7 generates multiple prediction boxes for each detected target and traditionally employs the Non-Maximum Suppression (NMS) algorithm to retain the most relevant ones. NMS ranks the prediction boxes based on their confidence scores and iteratively selects the box with the highest score, suppressing others that exhibit significant overlap—quantified by the Intersection over Union (IOU)—with the selected box. However, this approach considers only spatial overlap and ignores target shape, orientation, and contextual information. Additionally, the high computational complexity of NMS can impair inference speed in real-time applications. A further limitation lies in its sensitivity to the IOU threshold: a low threshold may erroneously eliminate boxes corresponding to distinct but adjacent targets, whereas a high threshold may retain redundant boxes for the same target, reducing detection precision.

To overcome these limitations, this study adopted the Soft-NMS algorithm [28] to optimize prediction box selection. Unlike standard NMS, Soft-NMS does not discard overlapping boxes outright. Instead, it applies a decay function to reduce their confidence scores proportionally to their IOU values—where greater overlap results in a steeper reduction. This strategy incorporates both spatial overlap and confidence levels, improving the model’s ability to distinguish between densely packed or overlapping white blood cells. Furthermore, Soft-NMS employs a confidence threshold to remove low-probability predictions, ensuring that only high-confidence boxes are preserved. This enhances classification accuracy by reducing missed detections due to occlusions. The pseudocode for the Soft-NMS algorithm is shown in Table 3.

The pseudocode for Soft-NMS is shown in Table 3. In the above code, when the highest confidence bounding box’s IOU exceeds a certain threshold with other prediction boxes, the prediction box is directly discarded by NMS. Soft-NMS, however, adjusts the confidence of overlapping boxes by setting a threshold and calculating the product of a weight adjustment function and the prediction box’s confidence to generate a new confidence score, allowing more flexible control over the output prediction boxes. The improved Soft-NMS algorithm yields better results when dealing with overlapping white blood cells. Compared to the traditional NMS algorithm, Soft-NMS retains more overlapping white blood cell prediction boxes, preventing both missed detections and false positives. This not only improves the accuracy of white blood cell classification but also enhances the overall performance and reliability of the classification system.

## 4. Experiment and Results

### 4.1. Experimental Environment and Configuration

This experiment used Python 3.8 as the code compilation version and Pytorch 1.13.1 as the deep learning framework. The code debugging was conducted in a Windows 10 environment. To shorten the model training time, the training was performed on a cloud server. The experimental setup included an AMD EPYC 7542 processor, a 3090 GPU (24 GB VRAM), and 127 GB of RAM. All experiments were implemented using Pytorch 1.13.1 and CUDA 11.7.0. The detailed experimental environment is shown in Table 4.

In this experiment, the pre-trained weight file used was changed to yolov7.pt in the yolov7-F/train.py file. The image input size was set to 640 × 640, and the data loading configuration file was set as Fyolov7.yaml. The hyperparameters are shown in Table 5.

### 4.2. Performance Evaluation Indicators

The performance of a classification model is generally evaluated from three dimensions: model size, computational complexity, and detection accuracy. In this paper, to comprehensively assess the model’s performance, three metrics were chosen: parameters, GFLOPS, and mean average precision (MAP).

MAP refers to the average accuracy across all classes, calculated by averaging the average precision (AP) of multiple classes. A higher MAP value indicates better average precision across all categories. GFLOPS measures the model’s computational complexity, indicating the number of floating-point operations (FLOPs) the model performs during the forward inference process. A higher GFLOPS value generally corresponds to higher computational load; in this scenario, as the number of floating-point operations increases, the model’s inference speed may decrease. Parameters represents the total number of parameters with weights across all layers in the model. A larger parameter count typically results in longer training and inference times. Furthermore, a larger number of parameters also increases the model’s computational load, which can negatively affect the running speed. These three metrics provide a comprehensive way to evaluate the model’s trade-offs between accuracy, computational efficiency, and model size.

### 4.3. Experimental Results and Analysis

#### 4.3.1. Analysis of the Results of the Improved Backbone Network

To analyze the impact of different backbone networks on the performance of the CCE-YOLOv7 model, comparative experiments were conducted using seven backbone networks, including QARepVGG (Quantized Attention Representational VGG), Swin Transformer (Shifted Window Transformer), EffQAFPN (Efficient Quantized Attention Feature Pyramid Network), and GhostV2 (GhostNet V2). As shown in Table 6, Conv2Former (Convolutional Transformer) achieved the highest MAP of 85.1% and a parameter count of 34.4 M, which was reduced by 2.1 M compared to the original model, and a computational complexity of 97.4 GFLOPS (Giga Floating Point Operations Per Second), which was reduced by 6.5 GFLOPS, resulting in a 6.2% improvement over the original network. Among the other six backbone strategies, RepGhost (Representation Ghost) led to a 3% decrease in MAP compared to the original model, while Swin Transformer increased the MAP by 2.3% but exhibited a computational complexity of 118.6 GFLOPS, which was 14.7 GFLOPS higher than the original model. In contrast, Conv2Former enhanced the capability of the CCE-YOLOv7 white blood cell classification network in capturing global contextual information from images, significantly improving classification performance. In summary, adopting Conv2Former as the backbone network effectively enhanced the classification accuracy of the CCE-YOLOv7 model while reducing both parameter count and computational complexity, achieving a balance between efficiency and lightweight design.

#### 4.3.2. Analysis of the Results of Improve Sampling

To further investigate the impact of the $K_{encoder}$ and $K_{up}$ parameters in the CARAFE algorithm on the performance of the CCE-YOLOv7 model, extensive experiments were conducted on the white blood cell classification dataset, with the results summarized in Table 7. The dataset provides information on model performance, parameter count, and computational complexity. To ensure that the content encoder has a sufficiently large receptive field for accurately predicting larger reconstruction kernels, an increase in $K_{up}$ requires a corresponding increase in $K_{encoder}$ Expanding the receptive field helps the model capture richer contextual information, thereby enhancing the feature MAP’s information extraction capability.

The experimental results indicate that simultaneously increasing both $K_{encoder}$ and $K_{up}$ can enhance the model’s performance. If only one of these parameters is increased, the model’s performance does not improve significantly. Moreover, the size of the upsampling kernel is closely related to the receptive field, with larger kernel sizes corresponding to larger receptive fields, thereby enabling more effective feature extraction from feature MAPs. When $K_{encoder}$ and $K_{up}$ were set to 5 and 7, respectively, the model achieved the highest MAP value of 87.3%. However, at this setting, the model’s parameter count reached 34.7 M, and its computational complexity increased to 95.5 G, which negatively impacted the detection speed. Therefore, to balance detection accuracy and speed, a compromise was adopted by setting $K_{encoder}$ and $K_{up}$ in the CARAFE upsampling algorithm to 3 and 5, respectively. This configuration maintained the model’s detection accuracy to a certain extent while reducing complexity and computational load, ultimately achieving faster detection speed in practical applications.

#### 4.3.3. Analysis of Ablation Experiment Results

To assess the impact of the improved modules on the model’s overall performance, ablation experiments were conducted on the five proposed enhancement strategies: data augmentation, backbone network, feature fusion, upsampling algorithm, and non-maximum suppression algorithm. The results of these ablation experiments are presented in Table 8.

The ablation experiment results show that data augmentation increased the MAP accuracy by 1.1%. Introducing only the CARAFE algorithm and the EMA module improved the MAP accuracy by 2.6% and 3.1%, respectively. The significant performance improvement from adding only the EMA module is attributed to the enhanced channel attention, which better captures shallow semantic information. Introducing the CARAFE operator further increased the MAP accuracy by 1.6%, indicating a stronger ability to extract semantic information from feature MAPs. For the two modules that improved based on data augmentation, the combination of the Conv2Former and CARAFE modules increased the MAP accuracy by 5.2%, with the model’s parameter count reduced by 2.1 M to 34.4 M, and the computational load decreased by 6.3 G to 97.6 G. For the three improved modules, the combination of Conv2Former, CARAFE, and EMA modules led to a 7.4% increase in MAP accuracy. The combination of CARAFE and EMA modules significantly enhanced model performance. The CARAFE algorithm improved the ability to extract semantic information, while EMA optimized feature fusion, enhancing the model’s predictive capabilities. After introducing the Soft-NMS algorithm, the accuracy of the predicted bounding boxes improved. With no change in parameters or computational load, the model’s MAP increased by 0.4%, reaching 89.3%. Overall, the proposed strategies and optimization resulted in a 7.8% improvement in MAP accuracy, a reduction of 2 M in parameter count, and a 5.7 G reduction in computational load (to 98.2 G), fully validating the improved CCE-YOLOv7 model’s performance in white blood cell classification.

#### 4.3.4. Analysis of Comparative Experimental Results

To comprehensively evaluate the classification performance and effectiveness of the improved CCE-YOLOv7 model, both quantitative and qualitative analyses of the experimental results were conducted. Additionally, the improved model was compared with other commonly used classification models, which were trained and validated under the same configuration and using the same dataset in order to provide a thorough comparison of classification performance.

**Figure 8:**
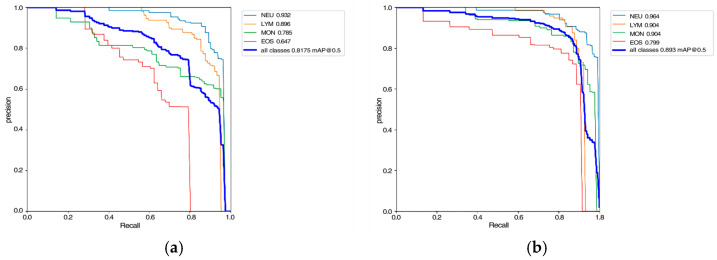
P-R curves of YOLOv7 and CCE-YOLOv7; (**a**) P-R curve for the YOLOv7 model; (**b**) P-R curve for the CCE-YOLOv7 model.

Quantitative Results Analysis: To objectively assess the classification performance of the improved CCE-YOLOv7 model on the white blood cell image dataset, a comparison with the pre-improvement version was conducted. The experimental results are illustrated in Figure 8, which presents the precision–recall (P-R) curves for each class. The P-R curve evaluates the trade-off between precision (*y*-axis, representing classification accuracy for positive predictions) and recall (*x*-axis, indicating the model’s ability to detect all relevant instances). A higher area under the curve (AUC) corresponds to superior classification performance. In the figure, “NEU” denotes neutrophils, “LYM” represents lymphocytes, “MON” indicates monocytes, and “EOS” corresponds to eosinophils.

As depicted in Figure 8, the model’s precision remained stable with increasing recall initially, followed by a gradual decline. When recall approached 1 (indicating comprehensive detection of all relevant instances), precision deteriorated sharply. The results demonstrate that, compared to the original YOLOv7 network, the improved model exhibits significant enhancements in classifying the four white blood cell types, particularly in sustaining higher precision at elevated recall levels. This underscores the improved CCE-YOLOv7 model’s robustness and reliability in white blood cell classification tasks.

**Figure 9:**
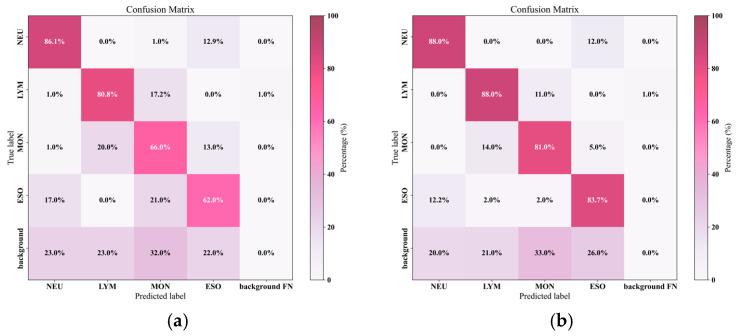
Confusion Matrix Comparison of YOLOv7 and CCE-YOLOv7; (**a**) confusion matrix for the YOLOv7 model; (**b**) confusion matrix for the CCE-YOLOv7 model.

To more intuitively illustrate the classification performance differences between YOLOv7 and CCE-YOLOv7 for the four types of white blood cells, confusion matrix plots for both models were generated, as shown in Figure 9. The confusion matrix clearly demonstrates the classification capabilities of the two models for white blood cells. It represents the model’s ability to correctly predict each class and offers insight into the types of errors the model makes. In the confusion matrix, columns represent predicted classes, rows represent true classes, and diagonal elements reflect correct classifications, while non-diagonal elements indicate misclassifications. The dark-colored blocks along the diagonal reflect the proportion of correctly predicted classes. As shown in Figure 9a, the model’s classification probabilities for MON and EOS were 66% and 62%, respectively. In Figure 9b, the model’s classification probabilities for MON and EOS were 81% and 83.7%, respectively. This clearly shows that the improved CCE-YOLOv7 algorithm achieved a higher classification accuracy, yielding superior results.

**Figure 10:**
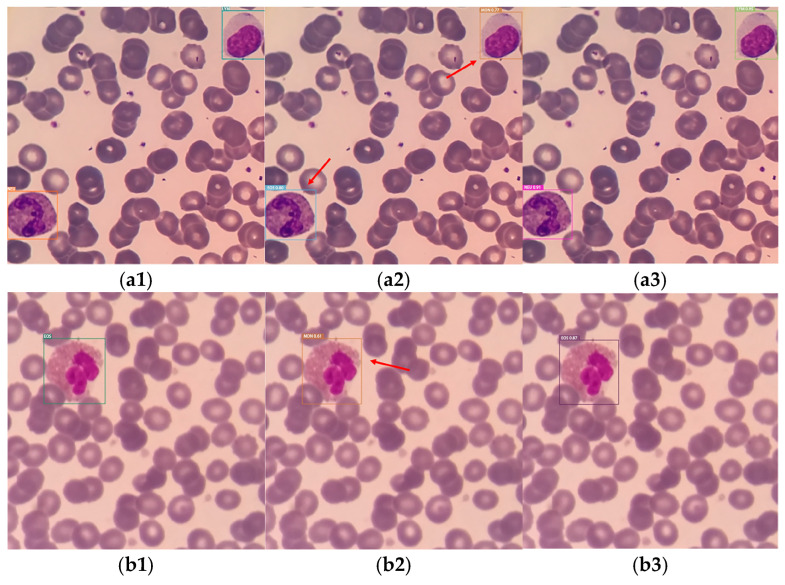

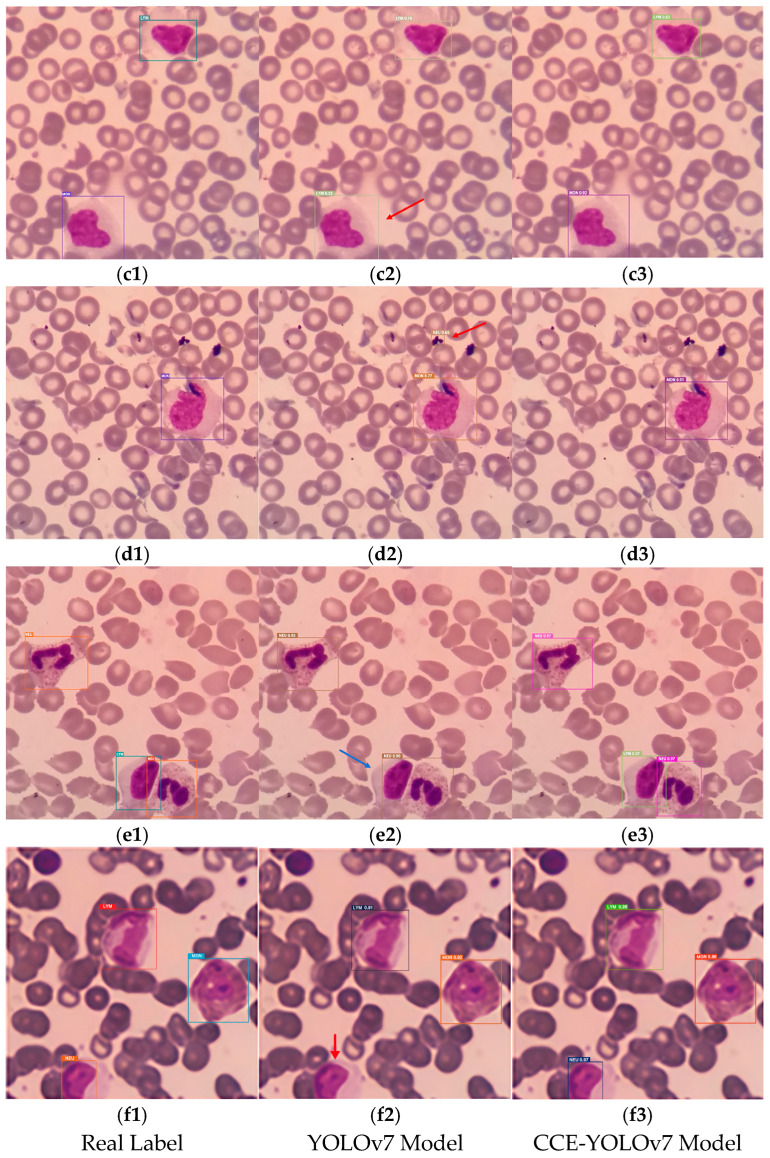
Leukocyte classification test results; (**a1**–**f1**) is the real label; (**a2**–**f2**) is the result detected by the YOLOv7 model; (**a3**–**f3**) is the result detected by the CCE-YOLOv7 model.

Qualitative Results Analysis: To visually examine the actual performance of the improved CCE-YOLOv7 model in white blood cell classification, five typical white blood cell samples with distinct characteristics were selected. In each row, the first column shows the true label of the sample, while the second and third columns display the classification detection results of the YOLOv7 model and CCE-YOLOv7 model, respectively, as shown in Figure 10. The blue arrows in the figure indicate missed detections, while the red arrows indicate false detections.

Figure 10 shows that (Figure 10(a2)) the YOLOv7 model incorrectly classified neutrophils and lymphocytes as eosinophils and monocytes, whereas the improved CCE-YOLOv7 model provided correct predictions. This misclassification occurred due to the subtle differences between the nuclei and cytoplasm of different white blood cell types, leading to errors. In Figure 10(b1–b3,c1–c3), it can be seen that the original YOLOv7 model also made classification errors, whereas the improved model correctly predicted the results. In Figure 10(d1), it is shown that the true label of the sample contained only one monocyte, while in Figure 10(d2), it can be seen that one neutrophil was detected, erroneously classifying the staining liquid used in sample preparation as a neutrophil. In Figure 10(e2), a lymphocyte was missed by the YOLOv7 model, but the improved CCE-YOLOv7 model did not miss any cells and accurately predicted all white blood cells to be classified. In Figure 10(f2), the YOLOv7 model missed one neutrophil, but the improved CCE-YOLOv7 model did not miss any cells and accurately predicted all leukocytes in their classifications. Additionally, Figure 10 shows that the confidence levels of the CCE-YOLOv7 model’s predictions were consistently higher than those of the YOLOv7 model. This is due to the improved feature fusion and backbone network, which allowed the network to capture both local and global information in the white blood cell images, resulting in higher classification confidence.

To further validate the advantages of the improved CCE-YOLOv7 model in white blood cell image classification, comparison experiments were conducted with commonly used classification models, such as the SSD model, Faster-RCNN model, and YOLOv4 model, under the same experimental conditions and parameter configurations. The performance analysis was conducted on the experimental results of different models, including parameters, computational load, and MAP values, to verify the superiority of the CCE-YOLOv7 model. The results are shown in Table 9.

Compared to the YOLOv7 model, the proposed CCE-YOLOv7 demonstrated significant improvements in mean average precision (MAP) across four white blood cell (WBC) categories. Specifically, MAP increased by 3.2% for neutrophils (NEU), 0.8% for lymphocytes (LYM), 11.9% for monocytes (MON), and 15.2% for eosinophils (EOS), resulting in an average MAP improvement of 7.8% across all categories. Furthermore, the model achieved enhanced computational efficiency with a reduction of 2 million parameters and 5.7 GFLOPs in computational load. While YOLOv5s exhibited the lowest computational complexity (7.1 M parameters and 16.5 GFLOPs), its overall MAP of 80.5% remained substantially lower than that of CCE-YOLOv7. The proposed model also outperformed other mainstream detectors in both accuracy and efficiency. Compared to SSD, Faster R-CNN, YOLOv4, and YOLOv5l, CCE-YOLOv7 reduced computational load by 173.6 GFLOPs, 303.6 GFLOPs, 21.5 GFLOPs, and 10.9 GFLOPs respectively, while improving MAP by 22.5%, 17.2%, 11.7%, and 7.4%. In comparison with contemporary architectures, CCE-YOLOv7 showed superior performance over YOLOX with a 6.7% MAP improvement for WBC classification, alongside reductions of 19.7 M parameters and 57.4 GFLOPs. When compared to YOLOv6, it achieved an 8.3% higher MAP while using 0.4 M fewer parameters. The model also maintained advantages over newer versions: versus YOLOv8, it improved overall MAP by 2.0% while reducing parameters by 1.7 M and computational load by 7.4 GFLOPs; compared to YOLOv11, it achieved a 4.6% mAP increase with 0.7 M fewer parameters and 1.5 GFLOPs less computation.

These comprehensive comparisons demonstrate that CCE-YOLOv7 achieved state-of-the-art performance in WBC image classification, effectively balancing high detection accuracy with enhanced computational efficiency. This dual optimization makes the model particularly suitable for medical imaging applications requiring both precision and resource efficiency.

## 5. Summary

This study addressed the challenges arising from the subtle feature differences among various types of white blood cells, small convolutional receptive fields, and the insufficient utilization of local context information. A white blood cell classification algorithm based on CCE-YOLOv7 was thus herein proposed. First, the main improvement strategies were introduced, focusing on optimizations to four components: the backbone network, upsampling algorithm, feature fusion, and non-maximum suppression algorithm. Next, the parameter settings, experimental environment, and performance evaluation metrics were clearly outlined. Ablation experiments were performed to examine the impact of each module on model performance. Finally, both quantitative and qualitative analyses of the experimental results were provided. A comprehensive comparison with other commonly used models under identical configuration conditions was conducted, demonstrating the significant advantages of the CCE-YOLOv7 model in white blood cell image classification tasks.

## Figures and Tables

**Table 1:** Pathological Features Caused by Deviations in White Blood Cell Population Concentrations [15].

White Blood Cell Subtype	(410) × 10^9^/L Normal Reference Range	Pathologic Features Accompanying Concentration Bias	
		Elevated	Decreased
Neutrophils (NEU)	50–70%	Severe tissue injury, hemorrhage, acute suppurative infections, encephalitis B, etc.	Influenza, infectious diseases, measles, etc.
Eosinophils (EOS)	0.5–5%	Allergic diseases, hematologic diseases, parasitic diseases, etc.	Taking hormonal drugs, myelodysplastic syndromes, typhoid fever, etc.
Basophils (BAS)	0.5–1%	Rheumatoid arthritis, diabetes mellitus, malignancy, myelofibrosis, etc.	Hyperthyroidism, Cushing’s syndrome, aplastic anemia, etc.
Lymphocytes (LYM)	20–40%	Pertussis, chronic lymphocytic leukemia, and viral infections;	Immunodeficiency, X-ray exposure, etc.
Monocytes (MON)	3–8%	Malaria, tuberculosis, etc.	-

**Table 2:** Basic Characteristics of Different Types of White Blood Cells.

White Blood Cell Categories	Cytoplasmic Diameter	Proportion	Nucleus	Cytoplasm
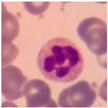 neutrophil	10–15 uL	About 50% to 70% of the total number of white blood cells	The nuclei are bluish-purple, rod-shaped, S-shaped, leaf-shaped, etc.	The cytoplasm is rich and full of neutral granules, which are fine and light blue in color.
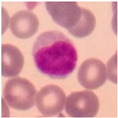 lymphocyte	6–15 uL	Accounts for approximately 20–40% of the total white blood cell count.	The nucleus is roughly round or oval and positioned to one side.	The cytoplasm is sparse or minimal, with purplish-red granules.
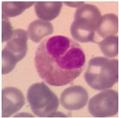 eosinophil	11–16 uL	Approximately 0.5% to 5% of the total white blood cell count.	The nucleus is lobulated or rod-shaped, with clumped chromatin and distinct parachromatin.	The cytoplasm is abundant, round or oval, and evenly distributed.
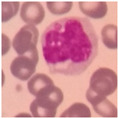 monocyte	12–20 uL	Accounts for approximately 3% to 8% of the total white blood cell count.	The nucleus is irregular and oval-shaped, with loose chromatin and no visible nucleolus.	The cytoplasm is abundant, light gray-blue, and slightly tinged with purplish-red.

**Table 3:** Soft-NMS algorithm pseudocode.

Input: initial set of detection frames $B=\left\{b_{1},b_{2},\ldots ,b_{n}\right\}$, set of detection scores $S=\{s_{1},s_{2},\ldots ,s_{n}$}; Output: filtered set of test boxes K;	
While: B≠∅	
$b_{n}=max(B)$	// $b_{n}$ highest confidence detection frame
$B=b-b_{n}$	Delete $b_{n}$ from B
$K=K+b_{n}$	Add $b_{n}$ to K
$for(b_{i}\in B)$	
$U_{i}=f(IoU(b_{n},b_{i}))$	Calculate the penalization factor
$s_{i}=\mu_{i}*s_{i}$	Confidence scores are updated
$if(s_{i}<\sigma )$	
$B=b-b_{i}$	Delete *b_i_* in *B* when less than the threshold *σ*
return K	0.5

**Table 4:** Experimental environment requirements.

Name	Parameter Description
Operating system	Windows10
CPU	AMD EPYC 7542
GPU	3090-24 G
Memory	127 G
CUDA version	11.7
Python	3.8
Pytorch	1.13.1

**Table 5:** Super parameter settings.

Name	Numerical Value
Image size	640 × 640 × 3
Batch size	4
lr0	0.01
lrf	0.1
Optimizer	SGD
Weight decay	0.0005
Momentum	0.937

**Table 6:** Experimental Comparison of Different Backbone Network Improvement Strategies.

Core Network	Quantity of Participants/M	Computational Volume/G	MAP/% (NEU)	MAP/% (LYM)	MAP/% (MON)	MAP/% (EOS)	MAP/% (ALL)
ELAN	36.5	103.9	95.1	91.0	78.0	66.5	82.6
QARepVGG	37.5	104.7	96.9	89.1	80.9	71.8	84.7
ConvNext	34.2	96.7	93.0	90.7	80.3	72.2	84.1
Swin Transformer	34.3	118.6	96.7	88.6	82.9	71.4	84.9
RepGhost	34.0	96.2	95.7	91.0	71.8	59.7	79.6
EffQAFPN	34.2	97.0	93.5	89.0	73.3	68.2	81.0
GhostV2	33.9	96.0	94.5	89.0	75.0	63.3	80.5
Conv2Former	34.4	97.4	94.0	89.9	83.7	72.6	85.1

**Table 7:** Effectiveness of model detection with different $K_{encoder}$ and $K_{up}$ values.

$K_{encoder}$	$K_{up}$	Quantity of Participants/M	Computational Volume/G	MAP/% (NEU)	MAP/% (LYM)	MAP/% (MON)	MAP/% (EOS)	MAP/% (ALL)
1	3	34.4	97.6	95.4	86.1	87.8	76.0	86.3
1	5	34.4	97.6	93.0	93.0	86.8	72.0	86.2
3	3	34.4	97.6	96.5	89.2	85.6	71.4	85.7
3	5	34.4	97.6	95.7	91.7	83.7	75.6	86.7
3	7	34.5	97.8	96.5	89.3	85.2	75.5	86.6
5	5	34.6	98.0	96.0	87.6	86.7	75.1	86.4
5	7	34.7	98.5	96.6	88.2	82.0	82.4	87.3

**Table 8:** Analysis of ablation experiment results.

Yolov7	Data Enhancement Strategy	Conv2Former	CARAFE	EMA	Soft-NMS	Quantity of Participants/M	Computational Volume/G	MAP/% (ALL)
√						36.5	103.9	81.5
√	√					36.5	103.9	82.6
√		√				34.4	97.4	83.8
√			√			36.5	104.1	84.1
√				√		36.5	104.5	84.6
√					√	36.5	103.9	81.8
√	√	√				34.4	97.4	85.1
√	√	√	√			34.4	97.6	86.7
√	√	√	√	√		34.5	98.2	88.9
√	√	√	√	√	√	34.5	98.2	89.3

The √ Indicates that the module is used in the current set of experiments.

**Table 9:** Experimental comparison of different network models.

Core Network	Quantity of Participants/M	Computational Volume/G	MAP/% (NEU)	MAP/% (LYM)	MAP/% (MON)	MAP/% (EOS)	MAP/% (ALL)
SSD	24.0	274.5	79.4	78.5	55.9	53.3	66.8
Faster-RCNN	136.8	401.8	82.6	79.0	72.5	54.6	72.1
YOLOv4	52.4	119.7	93.2	87.4	75.2	54.7	77.6
YOLOv5l	46.5	109.1	92.5	87.4	82.4	65.3	81.9
YOLOv5s	7.1	16.5	94.5	89.0	75.0	63.3	80.5
YOLOX	54.2	155.6	95.1	91.0	78.0	66.5	82.6
YOLOv6	34.9	85.8	93.5	89.0	73.3	68.2	81.0
YOLOv7	36.5	103.9	93.2	89.6	78.5	64.7	81.5
YOLOv8	36.2	105.6	95.4	90.1	80.1	74.5	87.3
YOLOv11	35.8	96.7	94.1	89.2	87.6	71.8	84.7
CCE-YOLOv7	34.5	98.2	96.4	90.4	90.4	79.9	89.3

## Data Availability

The dataset used in this study is the publicly available BCCD (Blood Cell Count and Detection) dataset, which can be freely accessed from the following repository: https://github.com/Shenggan/BCCD_Dataset (accessed on 12 March 2025).
